# Bioprosthetic vs mechanical mitral valve replacement for infective endocarditis in patients aged 50 to 69 years

**DOI:** 10.1002/clc.23407

**Published:** 2020-06-04

**Authors:** Xingjian Hu, Weiwei Jiang, Minghui Xie, Ruikang Guo, Wai Yen Yim, Nianguo Dong, Yin Wang

**Affiliations:** ^1^ Department of Cardiovascular Surgery, Union Hospital, Tongji Medical College Huazhong University of Science and Technology Wuhan China; ^2^ Department of Gastroenterology, Union Hospital, Tongji Medical College Huazhong University of Science and Technology Wuhan China; ^3^ Quality Control Center of Cardiovascular Surgery Health Committee of Hubei Province Wuhan China

**Keywords:** bioprosthesis, endocarditis, long‐term outcome, mechanical valve, mitral valve replacement

## Abstract

**Background:**

The optimal choice of the valve prosthesis in mitral valve replacement (MVR) for infective endocarditis (IE) is controversial and challenging, particularly for younger patients.

**Hypothesis:**

The postoperative outcomes of mechanical and biological MVR in IE patients aged 50 to 69 years are different.

**Methods:**

All IE patients aged 50 to 69 years with primary MVR in Hubei province hospitals from 2002 to 2018 were retrospectively reviewed. The median duration of follow‐up was 8.7 years (IQR, 6.8‐10.9 years). Propensity score matching (1:3 ratio) was used to yield 492 patients with comparable baseline features between bioprostheses and mechanical prosthetic valve groups. Outcomes were postoperative mid‐ to long‐ term survival, mitral valve reoperation, prosthetic valve endocarditis (PVE), stroke, and major bleeding events.

**Results:**

Fifteen‐year survival after MVR was 80.6% in the mechanical valve group and 69.3% in the bioprostheses group (HR 0.545, *P* = .040). The cumulative incidence of mitral valve reoperation was 8.8% with mechanical valves and 21.4% with bioprostheses (HR 0.260, *P* = .002). The cumulative incidence of PVE was 5.6% with mechanical valves and 7.2% with bioprostheses (HR 0.629, *P* = .435). The cumulative incidence of stroke was 12.9% with mechanical valves and 10.5% with bioprostheses (HR 1.217, *P* = .647). The cumulative incidence of major bleeding was 12.0% with mechanical valves and 6.75% with bioprostheses (HR 1.579, *P* = .268).

**Conclusions:**

Mechanical valve prostheses were associated with better survival, lower rates of reoperation compared with bioprostheses within 15 years after MVR in IE patients aged 50 to 69. These findings suggest mechanical valve prostheses may be a more reasonable alternative to bioprostheses in this patient group.

## 1 INTRODUCTION

Infective endocarditis (IE) is a life‐threatening disease associated with high mortality and morbidity.^1^ Valve repairs is preferred over valve replacement; however, mitral valve replacement (MVR) is the cornerstone treatment, especially in the presence of severe valvular destruction or large vegetation.^2^, ^3^ There is no consensus recommendation of either bioprostheses or mechanical valve in the setting of IE, comorbidities and preferences should be considered in making the decision for prosthetic valve selection. A few studies comparing mechanical and biological prostheses in IE patients lead to contradictory results.^4^, ^5^, ^6^ For the general population with mitral valve disease, the European Society of Cardiology suggested surgery with mechanical valves for patients under 65 year while bioprosthesis for patients older than 70 year^7^; the American Heart Association guidelines recommend a mechanical valve prosthesis in patients under the age of 50 and a bioprostheses valve prosthesis over the age of 70.^8^ Between these ages, recent consensus guidelines recommend an individualized approach to prosthesis selection based on patient factors and preferences.

To clarify the ideal choice of prosthesis in native mitral valve IE, we designed this study and compared long‐term survival, incidence of reoperation, prosthetic valve endocarditis (PVE), stroke, and major bleeding events between bioprostheses and mechanical MVR in IE patients aged 50 to 69 years.

## 2 PATIENTS AND METHODS

A retrospective cohort study comparing long‐term outcomes after MVR was performed with either a mechanical prosthetic or a bioprostheses valve using the Hubei cardiac surgery registration system, an administrative database in which all inpatient hospitalizations of cardiovascular surgery in Hubei province are recorded. All patients aged 50 to 69 years who underwent primary MVR in Hubei province from 1 January 2002 to 31 December 2018 were identified. The protocol of this study was approved by the Ethical Committee of Tongji Medical College affiliated with Huazhong University of Science and Technology (02/03/2017S221).

**FIGURE 1:**
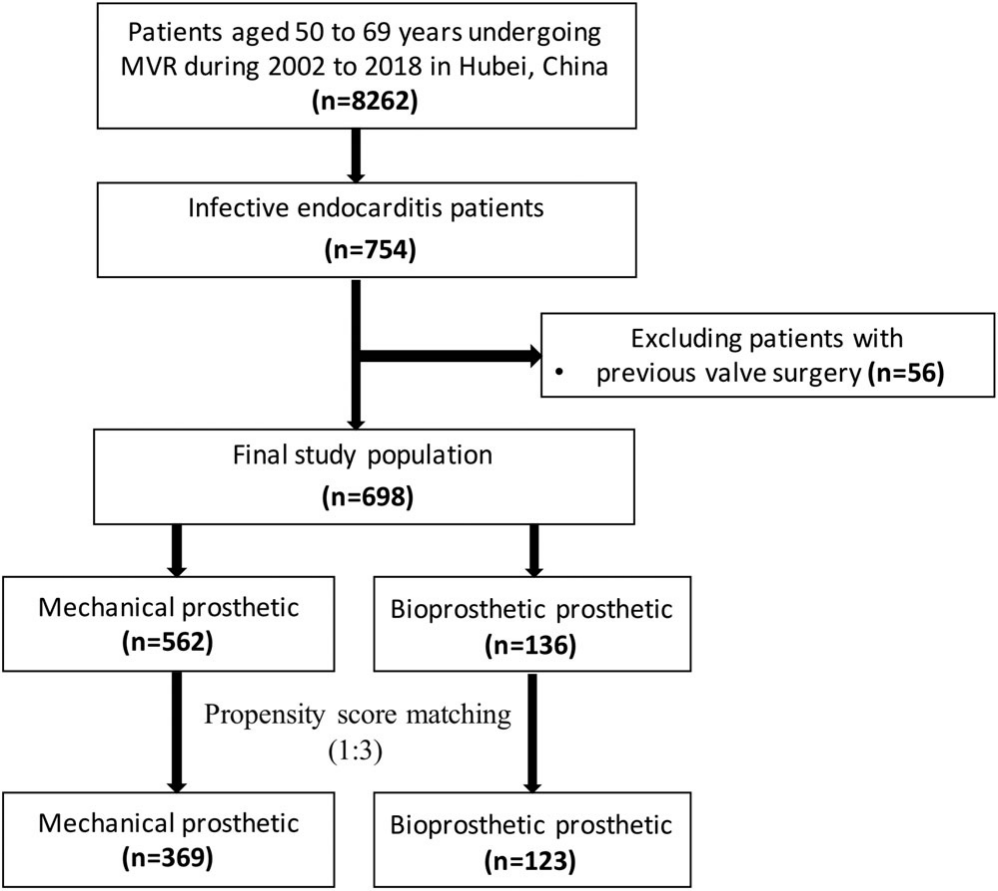
Study population flowchart

A total of 8262 patients aged 50 to 69 years who received MVR surgery between 1 January 2002 and 31 December 2018 were identified using International Classification of Diseases, Ninth Revision, Clinical Modification (ICD‐9‐ CM) procedure codes (35.23 and 35.24). Of these patients, 754 (9.13%) had a principal diagnosis of IE identified by ICD‐9‐CM diagnosis codes (421.0, 421.1, and 421.9) during the index hospitalization. Fifty six patients who had received previous valve surgery were excluded. Patients were more likely to undergo mechanical prosthetic MVR (562 patients; 80.5%) than bioprostheses prosthetic MVR (136 patients; 19.5%) in our study. To minimize potential selection bias, we calculated a propensity score from selected variables and matched each patient in the bioprostheses prosthetic group with each patient in the mechanical prosthetic group. Finally, 123 patients of the bioprostheses prosthetic group and 369 patients of the mechanical prosthetic group were identified and were eligible for analysis (Figure 1).

Preoperative clinical characteristics, operation variables, and postoperative inhospital complications were acquired according to the medical records. Follow‐up data were acquired through clinic reexamination and telephone interview. The follow‐up was 98.2% complete; median follow‐up time was 8.7 years (interquartile range [IQR], 6.8‐10.9 years) with maximum follow‐up of 16.5 years. Median follow‐up time was 8.8 years (IQR, 6.8‐11.0 years) in the mechanical prosthesis group compared with 8.5 years (IQR, 6.6‐10.8 years) in the bioprosthesis group.

Adverse events were classified according to the standardized definitions from the Society of Thoracic Surgeons/American Association for Thoracic Surgery “Guidelines for Reporting Morbidity and Cardiac Valvular Operations.”^9^ The primary study end‐points included overall mortality. The secondary study end‐points were reoperation, PVE, stroke, and major bleeding events. Stroke was defined as any cerebrovascular accident documented during the index hospitalization as well as any subsequent hospital admission in which the principal diagnosis was hemorrhagic or ischemic stroke. Reoperation was defined as any subsequent MVR. Major bleeding event was defined as requiring hospitalization or blood transfusion. PVE was diagnosed by ultrasonic cardiogram.

Baseline patient characteristics are represented as means with SD for continuous variables and proportions for categorical variables. To compare baseline differences in comorbidity between patients receiving mechanical prosthetic and bioprostheses valves, the *t* test was performed for continuous variables, the Pearson *χ* ^2^ test was performed for categorical variables, and standardized differences were calculated for all variables.

Confounding due to differences in baseline characteristics was addressed using propensity score matching.^10^ To calculate the propensity score, a hierarchical logistic regression model was fitted with bioprostheses implantation as the outcome. Covariates entered into the model include all measured baseline characteristics: age, sex, NYHA class III to IV, admission urgency, hypertension, diabetes mellitus, lung disease, liver disease, renal insufficiency, cerebrovascular disease, peripheral vascular disease, atrial fibrillation, concomitant tricuspid valve repair, concomitant coronary artery bypass grafting (CABG), urgent, or emergency status.

The area under the receiver operating characteristic curve for this model was 0.81. A 1:3 match was performed through a greedy algorithm based on local optimization using a propensity‐score caliper of width equal to 0.2.^11^ The baseline characteristics of the patient pairs matched by propensity score were compared using the paired *t* test for continuous variables and the McNamara test for categorical variables. Standardized difference that was less than 0.1 was deemed indicative of acceptable balance.^12^

For the primary end point, survival curves and 15‐year estimates were derived from Kaplan‐Meier method. For the secondary end points of reoperation, PVE, stroke, and major bleeding events, a competing risk analysis was performed to construct cumulative incidence function curves and to calculate 15‐year estimates. For all end points, marginal Cox proportional hazards regression models with robust sandwich variance estimators were fitted with only prosthesis type entered as a covariate. The difference in overall survival was compared using the Cox model, whereas the differences in secondary end points were evaluated using the Gray test. All data analyses were performed with the Statistical Package for Social Sciences 19.0 (SPSS, Inc., Chicago, IL). A *P* < .05 was considered statistically significant.

## 3 RESULTS

### 3.1 Patients characteristics

The baseline characteristics and operative characteristics of the overall cohort are presented in Table 1. Patients who underwent bioprostheses valve replacement were on average older (62.2 ± 4.1 vs 68.7 ± 5.2 years, *P* < .001), and more likely to have cardiovascular morbidity including hypertension (24.3% vs16.2%, *P* = .027), diabetes mellitus (21.3% vs13.2%, *P* = .016), liver disease (12.5% vs7.1%, *P* = .040), renal insufficiency(10.3% vs4.3%, *P* = .005). Patients who received mechanical prosthetic valves were more likely to have a history of atrial fibrillation (23.1% vs 14.7%, *P* = .032) and receive concomitant CABG (9.8% vs 4.4%, *P* = .046). A ratio of 1:3 propensity‐score matching produced 123 patient pairs. Age and all baseline comorbidities were balanced with the two groups (Table 1). There was no significant difference in 30‐day mortality (1.6% in the bioprosthesis group vs 2.4% in the mechanical prosthesis group, *P* = .651) after valve replacement.

**TABLE 1:** Patient baseline characteristics in the overall cohort and propensity score matching groups according to type of mitral valve replacement

		Overall				Propensity score matching			
	All patients (n = 698)	Bioprosthetic (n = 136)	Mechanical (n = 562)	Standardized difference, %	*P* value	Bioprosthetic (n = 123)	Mechanical (n = 369)	Standardized difference, %	*P* value
Age, mean (SD), y	60.3 (4.8)	62.2 (4.1)	58.7 (5.2)	20.2	<.001	60.6 (3.9)	59.9 (4.2)	2.7	.554
Male, n (%)	420 (60.2)	81 (59.6)	339 (60.3)	2.1	.871	77 (62.6)	237 (64.2)	5.3	.745
NYHA class III–IV, n (%)	140 (20.1)	32 (23.5)	108 (19.2)	4.9	.260	31 (25.2)	90 (24.4)	3.7	.856
Hypertension, n (%)	124 (17.8)	33 (24.3)	91 (16.2)	8.3	.027	30 (24.3)	75 (20.3)	4.1	.341
Diabetes mellitus, n (%)	103 (14.8)	29 (21.3)	74 (13.2)	11.7	.016	28 (22.8)	60 (16.3)	7.3	.103
Lung disease, n(%)	109 (15.6)	23 (16.9)	86 (15.3)	2.3	.643	20 (16.3)	78 (21.1)	7.8	.241
Liver disease, n (%)	57 (8.2)	17 (12.5)	40 (7.1)	11.1	.040	15 (12.2)	30 (8.1)	8.2	.175
Renal insufficiency, n (%)	38 (5.4)	14 (10.3)	24 (4.3)	14.3	.005	10 (8.1)	21 (5.7)	6.7	.340
Cerebrovascular disease, n (%)	59 (8.5)	11 (8.1)	48 (8.5)	4.5	.865	9 (7.3)	33 (8.9)	4.7	.576
Peripheral vascular disease, n (%)	15 (2.2)	4 (2.9)	11 (2.0)	2.1	.478	4 (3.3)	21 (5.7)	6.8	.286
Atrial fibrillation, n (%)	150 (21.5)	20 (14.7)	130 (23.1)	12.2	.032	18 (14.6)	81 (22.0)	5.6	.080
Concomitant tricuspid valve repair, n (%)	45 (6.5)	9 (6.6)	36 (6.4)	1.9	.928	8 (6.5)	27 (7.3)	1.6	.761
Concomitant CABG, n (%)	61 (8.7)	6 (4.4)	55 (9.8)	9.8	.046	6 (4.9)	33 (8.9)	2.2	.148
Urgent or emergency status, n (%)	24 (3.4)	4 (2.9)	20 (3.6)	3.5	.723	3 (2.4)	12 (3.3)	3.8	.650

Abbreviations: CABG, coronary artery bypass grafting; NYHA, New York Heart Association.

### 3.2 Survival

**FIGURE 2:**
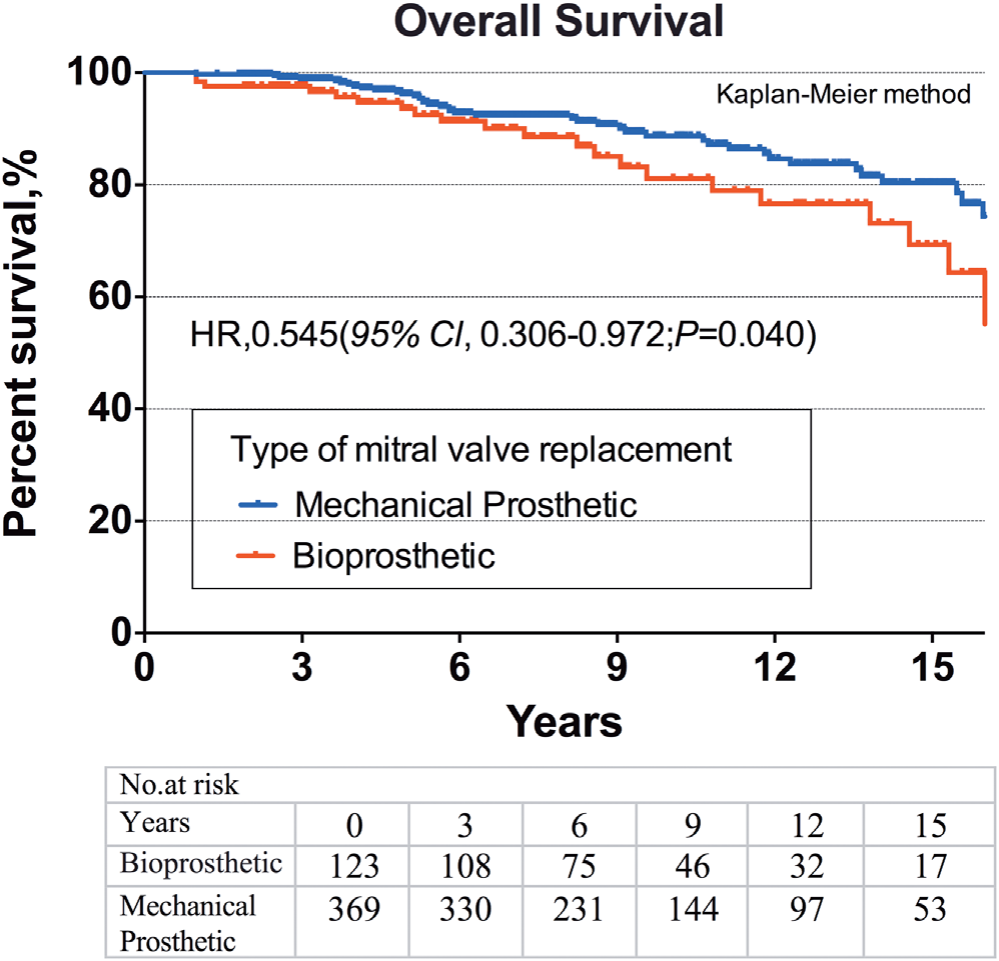
Fifteen‐year survival after mitral valve replacement for infective endocarditis patients aged 50 to 69 years according to prosthetic type: bioprostheses (red line) or mechanical (blue line)

Among patients matched by propensity score, mid‐ to long‐term survival was significantly higher among patients treated with a mechanical prosthetic than those treated with a biological prosthesis (hazard ratio [HR], 0.545 [95% CI, 0.306‐0.972], *P* = .040; Figure 2). A total of 42 (11.4%) deaths occurred in the mechanical prosthesis group and 22 (17.9%) deaths occurred in the bioprosthesis group, the actuarial survival at 1, 5, 10, and 15 years were 99.7%, 96.5%, 88.9%, and 80.6% in the mechanical prosthesis group, and 98.3%, 93.6%, 81.4%, and 69.3% in the bioprosthesis group, respectively.

### 3.3 Reoperation

**FIGURE 3:**
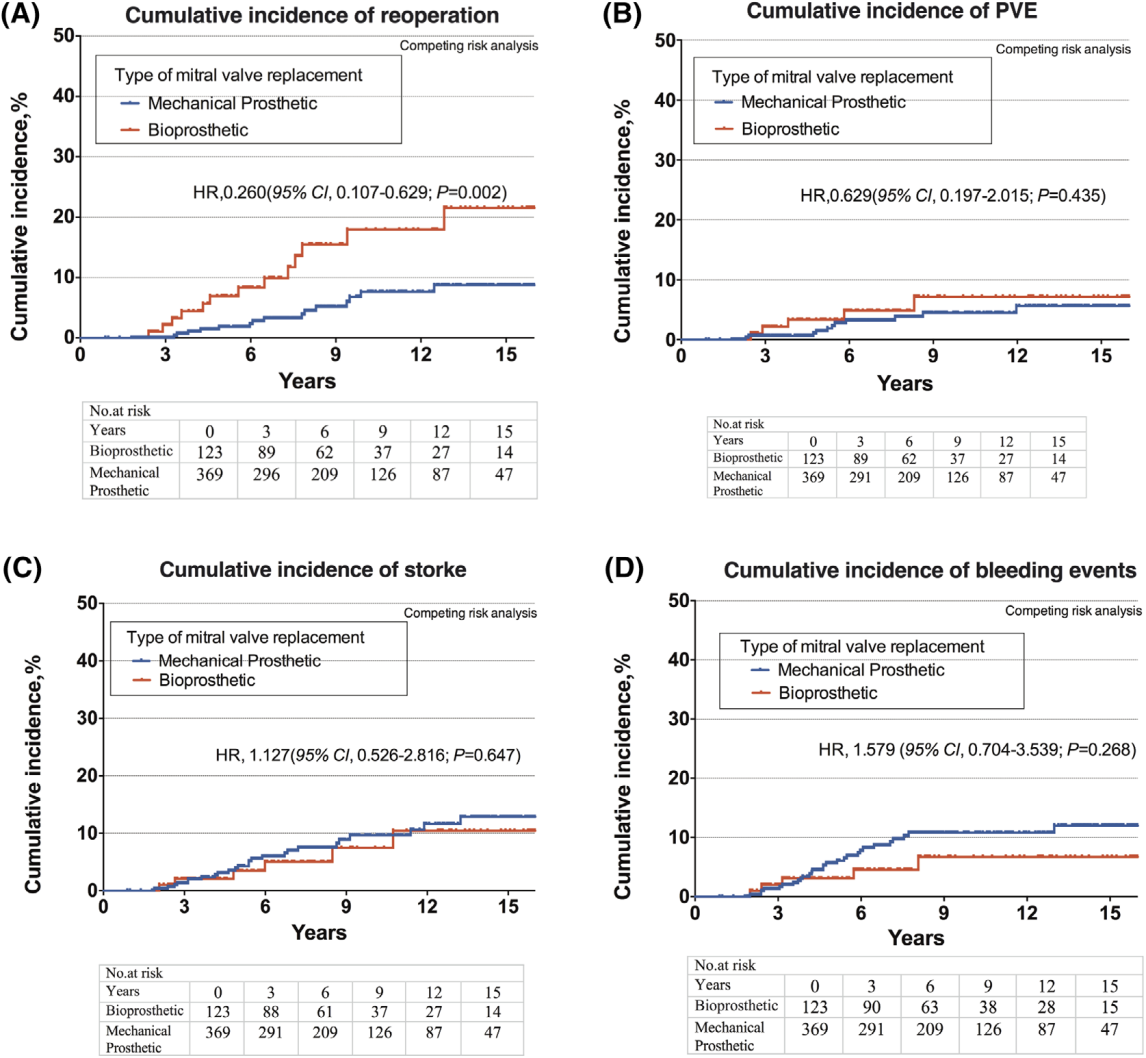
Cumulative Incidence of mid‐ to long‐term outcomes after mitral valve replacement for infective endocarditis patients aged 50 to 69 years according to prosthetic type: bioprostheses (red line) or mechanical (blue line). A, Reoperation. B, Prosthetic valve endocarditis. C, Stroke. D, Major bleeding events

A total of 28 patients received a reoperation in mitral valve position, and 13 patients in the bioprosthesis group while 15 patients in the mechanical prosthesis group. Overall 30‐day mortality of the reoperation was 10.7% (3/28). The cumulative incidence of reoperations was significantly lower among patients treated with a mechanical prosthetic than those treated with a biological prosthesis (HR, 0.260 [95% CI, 0.107‐0.629], *P* = .002; Figure 3A). The cumulative incidence of reoperations at 5, 10, and 15 years were 1.84%, 7.65%, and 8.76% in the mechanical prosthesis group, and 8.32%, 17.98%, and 21.54% in the bioprosthesis group, respectively.

### 3.4 Prosthetic valve endocarditis

A total of 11 PVE occurred in the mechanical prosthesis group and 5 PVE occurred in the bioprosthesis group. There was no significant difference in cumulative incidence of PVE between the mechanical prosthetic and bioprostheses group (HR, 0.629 [95% CI, 0.197‐2.015], *P* = .435; Figure 3B). The cumulative incidence of PVE at 5‐, 10‐, and 15‐years were 1.46%, 4.51%, and 5.60% in the mechanical prosthesis group, and 3.45%, 7.18%, and 7.18% in the bioprosthesis group, respectively.

### 3.5 Stroke

A total of 31 strokes occurred during follow‐up period, 25 in the mechanical prosthesis group and 6 in the bioprosthesis group. There was no significant difference of stroke between the two groups (HR, 1.217 [95% CI, 0.526‐2.816], *P* = .647; Figure 3C). Among the 31 patients of stroke, 11 patients were hemorrhagic and 20 were ischemic. Of the 20 ischemic strokes, 15 occurred in the mechanical prosthesis group and 5 occurred in the bioprosthesis group.

### 3.6 Major bleeding events

The rates of major bleeding events during the follow‐up period were not significantly different in patients with a mechanical and biological prosthesis (HR, 1.579 [95% CI, 0.704‐3.539], *P* = .268; Figure 3D). The cumulative incidence of major bleeding events at 5, 10, and 15 years were 5.67%, 10.82%, and 12.01% in the mechanical prosthesis group, and 3.11%, 6.75%, and 6.75% in the bioprosthesis group, respectively. Major bleeding was most commonly gastrointestinal (57.6%, 19/33), while 15 occurred in the mechanical prosthesis group and 4 occurred in the bioprosthesis group. Of all major bleeds, 36.4% (12/33) were intracranial with 11 in the mechanical prosthesis group and 1 in bioprosthesis group.

## 4 DISCUSSION

Despite the evolution of antimicrobial therapy and sepsis prevention, infections affecting the mitral valves continuously causing significant morbidity and mortality, leading to valvular malfunction, embolism, cerebrovascular events, and congestive heart failure. The surgery of mitral valve endocarditis is primarily determined by disease severity and valvular and annular destruction. For the lesion only limited to valve leaflets, mitral valve repairs is the preferred option since it shows better preservation of left ventricular function and low incidence of prosthesis‐related complications.^13^ However, IE with advanced valvular defects and paravalvular abscesses or fistulas require complete excision and MVR with artificial valve prosthesis.^14^

The dilemma about prosthesis selection will arise immediately once when patients decide to replace the destructed mitral valve.^15^ The choice of valve type is determined by balancing the risk of anticoagulation‐related and thromboembolic complications with mechanical valves vs the risk of structural failure and reoperation with bioprosthetic valves. Since there is no agreement on the optimal valve prosthetic choice in the setting of infective endocarditis, in most cases we need to refer to the consensus guidelines for the management of valvular heart disease.^16^, ^17^, ^18^ In general, the current guidelines on MVR (such as the American Heart Association (AHA)/American College of Cardiology (ACC) or the European Society of Cardiology (ESC) and the European Association for Cardio‐Thoracic Surgery (EACTS) guidelines) recommend using mechanical valve in for patients younger than 50 year and bioprosthesis for those who are older than 70 year. ^7^, ^8^, ^19^ However, the choice of mechanical or bioprosthetic valve in mitral position for patients aged 50 to 69 years is an uncharted territory in current guidelines.^15^, ^20^

Chikwe et al demonstrated no difference in survival for patients aged between 50 and 69 years requiring MVR, mechanical valves were associated with a significantly increased risk of stroke (HR 1.62; 95%CI: 1.10‐2.39) and bleeding event (HR 1.50; 95%CI: 1.05‐2.16), and a significant reduction in reoperation (HR 0.59; 95% CI: 0.37‐0.94).^20^ Kulik et al found similar results in MVR patients aged 50 to 65. There was no significant difference in late mortality, but an increase in the requirement for reoperation for bioprosthetic valves (HR 7.1; 95% CI: 1.8‐27.8) and an increased risk of thromboembolism for mechanical valves (HR4.1; 95% CI: 1.3‐12.7).^21^ In contrast, Goldstone et al demonstrated that receipt of a biologic prosthesis in mitral position was associated with significantly higher mortality than receipt of a mechanical prosthesis among those 50 to 69 years of age (HR 1.16; 95% CI, 1.04‐1.30).^22^

If the influence of IE was putting into consideration, the controversy will be even more complicated. Toyoda et al analyzed the database in California and New York from 1998 to 2010 and concluded that bioprosthetic and mechanical valves are associated with similar survival and freedom from endocarditis recurrence in the general population.^5^ Delahaye et al used data from International Collaboration on Endocarditis Prospective Cohort Study and found that biological valve replacement is independently associated with a higher inhospital and 1‐year mortality.^4^ Kyto et al demonstrated that the use of mechanical valve is associated with lower mid‐term mortality compared with bioprosthesis in patients with native‐valve IE aged <70 years.^23^ These studies neither differentiated the outcomes of valve replacement of IE in the mitral or aortic position nor aimed the specific patient cohort aged range from 50 to 69 years.

To our best knowledge, here we presented the first propensity score‐matched study of IE patients aged 50 to 69 with long‐term follow‐up data from the database of Hubei Medical Quality Control Center of Cardiovascular Surgery. As the geographical center of China, Hubei provides an ideal representative for its vast area (72.394 mile^2^), a large population (53.9 million), and ethnic diversity (55 ethnic groups). Based on our findings, the 15‐year survival of valve replacement in either mechanical or bioprosthesis group in this specific age range is satisfied (77.4% vs 66.4%, *P* = .481). The cumulative incidence of mitral valve reoperation at 15 years was significantly higher in the bioprosthesis group compared with the mechanical prosthesis group (15.8% vs 6.2%, *P* = .038). The high reoperation rate in bioprosthesis group may be attributed to the combination of bioprosthesis failure and endocarditis recurrence. There is no obvious difference in endocarditis recurrence rates between two artificial valve groups as observed in our study, which is 3.1% in mechanical and 5.1% in bioprosthesis group at 15 years. The endocarditis recurrence rate of our cohort is lower than those reported by other literature regardless of the type of prosthetic valve,^24^, ^25^, ^26^ which can be explained by the patient characteristics, such as less dialysis dependence, intravenous drug abuse, and the type of pathogen. These factors are proved to be the main independent predictors of recurrent endocarditis.^5^, ^27^ Complications associated with anticoagulation, such as stroke and major bleeding events, are still higher in mechanical groups as usual.

Another thing worth mention is the reason why valve replacement for heart valve disease including endocarditis is still more preferred in China. The first possible explanation is the epidemiology difference between Chinese and Euro‐American patients.^28^ Overall, there are lesser IE patients with a history of intravenous drug abuse, dialysis‐dependent renal failure, cardiac implantable electrophysiological devices, or immunodeficiency in China. Besides, the majority of IE patients in China often have a long history of substandard oral antibiotics and antipyretics usage for a nonspecific fever –the classic sign of endocarditis, which makes them miss the best opportunity for valve repair since their severe valvular destruction. It is quite common in the clinic to encounter Chinese IE patients with large or mobile vegetations, valve dehiscence or penetration, and severely impaired heart function. Moreover, Chinese patients have more concerns about the durability of mitral valve repair and the possibility of recurrent infection due to incomplete resection of the infected tissue, which makes them prefer to select valve replacement.^29^

The main limitations of this study are its retrospective observational nature, which may impact generalizability. We use the propensity score analysis method to minimize the impact of confounders related to treatment selection and heterogeneity in baseline factors; however, the fact that some patients had contraindications for a long‐term anticoagulant therapy was not avoided in the present study and a randomized prospective trial should be designed to confirm our result and allow for a class IA recommendation. Second, the dataset does not contain information on the prosthesis model, including bovine vs porcine, homograft vs allograft, or duration and type of antibiotic therapy, which may affect the function and durability of the prosthesis. Third, there was no complete data regarding the etiology of the infective endocarditis and we failed to compare the etiology between the two groups. Fourth, the risk of recurrence and reoperation for patients admitted to hospitals out of Hubei province during the follow‐up period were not captured, and we may therefore have underestimated the number of events.

## 5 CONCLUSIONS

This propensity score‐matched study compared 15‐year outcomes between mechanical and bioprosthetic MVR in infective endocarditis patients aged 50 to 69 years. We found that there was no significant difference in incidence of prosthetic valve endocarditis, major bleeding, or stroke between the two types of prostheses, but mechanical valve prostheses were associated with the better survival and lower rate of reoperation. These findings suggest mechanical valve prostheses may be a more reasonable alternative to bioprostheses in this patient group.

## CONFLICT OF INTEREST

The authors declare no potential conflict of interests.

## References

[1] Vincent LL , Otto CM . Infective endocarditis: update on epidemiology, outcomes, and management. Curr Cardiol Rep. 2018;20(10):86. 30117004 10.1007/s11886-018-1043-2

[2] Lee HA , Cheng YT , Wu VC , et al. Nationwide cohort study of mitral valve repair versus replacement for infective endocarditis. J Thorac Cardiovasc Surg. 2018;156(4):1473‐1483. 29843917 10.1016/j.jtcvs.2018.04.064

[3] Toyoda N , Itagaki S , Egorova NN , et al. Real‐world outcomes of surgery for native mitral valve endocarditis. J Thorac Cardiovasc Surg. 2017;154(6):1906‐1912. 28942975 10.1016/j.jtcvs.2017.07.077

[4] Delahaye F , Chu VH , Altclas J , et al. One‐year outcome following biological or mechanical valve replacement for infective endocarditis. Int J Cardiol. 2015;178:117‐123. 25464234 10.1016/j.ijcard.2014.10.125

[5] Toyoda N , Itagaki S , Tannous H , Egorova NN , Chikwe J . Bioprosthetic versus mechanical valve replacement for infective endocarditis: focus on recurrence rates. Ann Thorac Surg. 2018;106(1):99‐106. 29452115 10.1016/j.athoracsur.2017.12.046

[6] Kyto V , Ahtela E , Sipila J , Rautava P , Gunn J . Mechanical versus biological valve prosthesis for surgical aortic valve replacement in patients with infective endocarditis. Interact Cardiovasc Thorac Surg. 2019;29(3):386‐392. 31121026 10.1093/icvts/ivz122

[7] Falk V , Baumgartner H , Bax JJ , et al. 2017 ESC/EACTS guidelines for the management of valvular heart disease. Eur J Cardiothorac Surg. 2017;52(4):616‐664. 29156023 10.1093/ejcts/ezx324

[8] Nishimura RA , Otto CM , Bonow RO , et al. 2017 AHA/ACC focused update of the 2014 AHA/ACC guideline for the Management of Patients with Valvular Heart Disease: a report of the American College of Cardiology/American Heart Association task force on clinical practice guidelines. J Am Coll Cardiol. 2017;70(2):252‐289. 28315732 10.1016/j.jacc.2017.03.011

[9] Akins CW , Miller DC , Turina MI , et al. Guidelines for reporting mortality and morbidity after cardiac valve interventions. J Thorac Cardiovasc Surg. 2008;135(4):732‐738. 18374749 10.1016/j.jtcvs.2007.12.002

[10] D’Agostino RB Jr . Propensity scores in cardiovascular research. Circulation. 2007;115(17):2340‐2343. 17470708 10.1161/CIRCULATIONAHA.105.594952

[11] Austin PC , Mamdani MM . A comparison of propensity score methods: a case‐study estimating the effectiveness of post‐AMI statin use. Stat Med. 2006;25(12):2084‐2106. 16220490 10.1002/sim.2328

[12] Austin PC , Schuster T . The performance of different propensity score methods for estimating absolute effects of treatments on survival outcomes: a simulation study. Stat Methods Med Res. 2016;25(5):2214‐2237. 24463885 10.1177/0962280213519716 PMC5051602

[13] Wang A , Gaca JG , Chu VH . Management considerations in infective endocarditis: a review. JAMA. 2018;320(1):72‐83. 29971402 10.1001/jama.2018.7596

[14] Toyoda N , Chikwe J , Itagaki S , Gelijns AC , Adams DH , Egorova NN . Trends in infective endocarditis in California and New York state, 1998‐2013. JAMA. 2017;317(16):1652‐1660. 28444279 10.1001/jama.2017.4287 PMC5470417

[15] Nishimura RA , Gentile F , Bonow RO . Guideline update on evaluation and selection of prosthetic valves. JAMA Cardiol. 2018;3(3):260‐261. 29365033 10.1001/jamacardio.2017.5123

[16] Bedeir K , Reardon M , Ramlawi B . Infective endocarditis: perioperative management and surgical principles. J Thorac Cardiovasc Surg. 2014;147(4):1133‐1141. 24412256 10.1016/j.jtcvs.2013.11.022

[17] Byrne JG , Rezai K , Sanchez JA , et al. Surgical management of endocarditis: the society of thoracic surgeons clinical practice guideline. Ann Thorac Surg. 2011;91(6):2012‐2019. 21620012 10.1016/j.athoracsur.2011.01.106

[18] Newton S , Hunter S . What type of valve replacement should be used in patients with endocarditis? Interact Cardiovasc Thorac Surg. 2010;11(6):784‐788. 20713537 10.1510/icvts.2010.234450

[19] Wang Y , Chen S , Shi J , Li G , Dong N . Mid‐ to long‐term outcome comparison of the Medtronic Hancock II and bi‐leaflet mechanical aortic valve replacement in patients younger than 60 years of age: a propensity‐matched analysis. Interact Cardiovasc Thorac Surg. 2016;22(3):280‐286. 26675564 10.1093/icvts/ivv347 PMC4986564

[20] Chikwe J , Chiang YP , Egorova NN , Itagaki S , Adams DH . Survival and outcomes following bioprosthetic vs mechanical mitral valve replacement in patients aged 50 to 69 years. JAMA. 2015;313(14):1435‐1442. 25871669 10.1001/jama.2015.3164

[21] Kulik A , Bedard P , Lam BK , et al. Mechanical versus bioprosthetic valve replacement in middle‐aged patients. Eur J Cardiothorac Surg. 2006;30(3):485‐491. 16857373 10.1016/j.ejcts.2006.06.013

[22] Goldstone AB , Chiu P , Baiocchi M , et al. Mechanical or biologic prostheses for aortic‐valve and mitral‐valve replacement. N Engl J Med. 2017;377(19):1847‐1857. 29117490 10.1056/NEJMoa1613792 PMC9856242

[23] Kytö V , Sipilä J , Ahtela E , Rautava P , Gunn J . Mechanical versus biological prostheses for surgical aortic valve replacement in patients aged 50–70. Ann Thorac Surg. 2019;S0003‐4975;19:31765‐31765. 10.1016/j.athoracsur.2019.10.027 31786289

[24] Sheikh AM , Elhenawy AM , Maganti M , Armstrong S , David TE , Feindel CM . Outcomes of surgical intervention for isolated active mitral valve endocarditis. J Thorac Cardiovasc Surg. 2009;137(1):110‐116. 19154912 10.1016/j.jtcvs.2008.07.033

[25] Manne MB , Shrestha NK , Lytle BW , et al. Outcomes after surgical treatment of native and prosthetic valve infective endocarditis. Ann Thorac Surg. 2012;93(2):489‐493. 22206953 10.1016/j.athoracsur.2011.10.063

[26] Chauvette V , Comtois MO , Stevens LM , et al. Mid‐term outcomes in nonelderly adults undergoing surgery for isolated aortic valve infective endocarditis: results from two Canadian centers. Can J Cardiol. 2019;35(11):1475‐1482. 31679619 10.1016/j.cjca.2019.06.027

[27] Krajinovic V , Ivancic S , Gezman P , Barsic B . Association between cardiac surgery and mortality among patients with infective endocarditis complicated by sepsis and septic shock. Shock. 2018;49(5):536‐542. 28991051 10.1097/SHK.0000000000001013

[28] Wang Y , Jiang WW , Dong NG . Clinical application of bioprosthesis in China: current status and future. Curr Med Sci. 2019;39(4):523‐525. 31346985 10.1007/s11596-019-2068-5

[29] Wang Y , Chen S , Hu XJ , Shi JW , Dong NG . Mid‐ to long‐term clinical outcomes of Hancock II bioprosthesis in Chinese population. Chin Med J. 2015;128(24):3317‐3323. 26668146 10.4103/0366-6999.171424 PMC4797507

